# Histo-ELISA technique for quantification and localization of tissue components

**DOI:** 10.1038/s41598-020-76950-1

**Published:** 2020-11-16

**Authors:** Zhongmin Li, Silvia Goebel, Andreas Reimann, Martin Ungerer

**Affiliations:** Advancecor GmbH, 82152 Martinsried, Germany

**Keywords:** Biological techniques, Immunology, Medical research

## Abstract

A novel Histo-ELISA technique is intended to facilitate quantification of target tissue proteins in a tissue section and involves the selection of target regions in the tissue section, application of streptavidin-conjugated HRP (horseradish peroxidase), coupled with peroxidase substrate—TMB (3,3′,5,5′-tetramethylbenzidine), and staining dye evaluation with ELISA reader. The target protein content (weight per volume unit) was translated from optical densities by a reference standard curve, obtained via parallel staining of the targeted protein-coated slides. To validate the technique, we carried out quantifications of IgG extravasation in ischemic and nonischemic brain sections in a mouse stroke model. With those obtained data and the reference of immunohistochemistry scores assessed on the adjacent sections, accuracy, sensitivity, and precision for the technique were evaluated. For all evaluated parameters, Histo-ELISA performance was either comparable to or better than the standard immunohistochemistry. A comparison with the data from the repeated measurements yielded a rather low coefficient of variation. The results confirmed that the technique is a fairly reliable quantitative test with rather high sensitivity, accuracy, precision, and reproducibility for detecting target protein content in tissue sections and that its tissue distribution and related subsequent morphological changes can be observed at the same time.

## Introduction

Immunohistochemistry (IHC) based methods are widely used to detect target proteins in different studies due to facts of ease of use, turnaround time, cost, and morphological information. However, traditional IHC is semi-quantitative at best in the sense of scientific strictness^1–5^. In addition, changes in chemicals, working temperature, section thickness, and reagent reaction duration can lead to a significant Lab-to-Lab or observer-to-observer variations on a classic IHC stain, which is reflected in the form of lower or higher staining intensity^6^.

Quantification of intra- or extra-cellar proteins in small pathological specimens is difficult with the other conventional methods. ELISA techniques or Western blotting often requires sample pooling to obtain a specimen sufficient for accurate analysis and lacks the profiles of relative tissue morphological change and target protein microscopic distribution. Accurate and robust measurement of target proteins in the intact tissues, without loss of morphological information, is a challenge.

In this article, we describe a Histo-ELISA technique, a fusion method of conventional ELISA, and standard ABC immunostaining to quantify and localize target proteins and to observe the related morphological changes concurrently. It is based on the application of peroxidase substrate—TMB (3,3′,5,5′-tetramethylbenzidine) instead of DAB (3,3′-diaminobenzidine) and ELISA reader evaluation at the end of classical immune staining. Histo-ELISA combines the ease, speed, and morphological information gained from classical IHC and a robust quantitative assessment of proteins in situ borne in conventional ELISA.

For the validation of the method, parameters such as sensitivity, limit, accuracy, precision, and repeatability have been assessed using quantification of Immunoglobulin (IgG) infiltration in a stroke mouse model. Level of IgG extravasation into the brain and related morphological changes are important indices to judge the degree of blood–brain barrier damage during cerebral ischemia^7–9^ and there are no methods currently available to do both in one intact tissue block at the same time. However, with the Histo-ELISA, we quantified IgG infiltration and observed pathological changes spontaneously and by the way, the effectiveness of revacept on the stroke was evaluated in the ischemia models.

## Materials and methods

The experiments, inclusive of tissue processing, cutting, staining, evaluation, and standard curve preparation, were finished in GLP laboratory, and the room temperature keeps at 24 °C.

### Tissue collection and preparation

All animal procedures were reviewed and approved by Oberbayern Animal Welfare Committee in Munich, Germany (Regierung von Oberbayern, Sachgebiet Tierschutz, reference number 55.2-1-54-2531-98-09). We confirm that all experiments were performed in accordance with relevant guidelines and regulations. Twelve 8–12 week old male C57Bl/6J mice, weighing 21 to 29 g, purchased from Charles River (Charles River, Sulzfeld, Germany) were randomly divided into 4 groups, 3 mice for each. These groups include saline-injected native mice (saline-intact), Revacept injected native mice (revacept-intact), saline-injected brain infarct mice (saline-infarct), and Revacept injected brain infarct mice (revacept-infarct).

For validation of Histo-ELISA technique, we evaluated IgG extravasation in brain sections after occlusion of the left middle cerebral artery (MCA). One hour ischemia was induced by placing a silicon-coated monofilament (Doccol, USA) in the MCA via the left common/internal carotid artery as described by Hata^10^. Left hemisphere ischemia of the brain was confirmed by flow reduction in the MCA, which was monitored with a laser Doppler flow probe attached to the left temporal skull.

**Figure 1:**
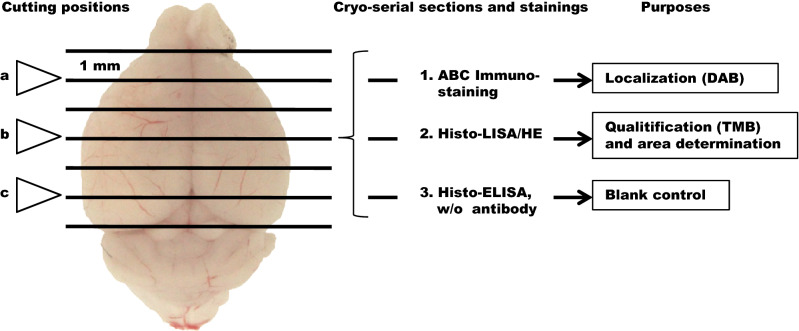
Experimental flowchart. Seven macro-sections of 1 mm in thickness were cut with brain slicer starting from the brain pole. The second (**a**), fourth (**b**) and sixth (**c**) were collected and embedded for farther cryo-serial sections. Three 10 µm thick cryo-serial sections were prepared for each macro-section and supposed to be for (1) ABC Immuno-staining colorized with DAB for the purpose of localization of interest proteins, (2) Histo-ELISA/HE colorized with TMB and the sequential HE staining, and (3) Histo-ELISA blank control without antibody application.

For determination of the effects of revacept on cerebral infarction with Histo-ELISA, revacept (1 mg/kg) or an equal volume of saline were injected respectively via the tail vein at the beginning of reperfusion. After 24 h of reperfusion, the mice were sacrificed, the brains were quickly removed and seven 1-mm-thick coronal sections were cut starting from the frontal pole using a mouse brain slice matrix (Cat BSM001.1, Zivic Lab Inc, USA), which had been soaked in ice-cold saline beforehand. We picked up the second, fourth, and sixth sections, with a 2 mm interval, for further histological cutting as described in Fig. 1. After undergoing optimum tissue compound (Tek OTC, VWR Chemicals, Leuven, Belgium) embedding and snap-frozen in a dry ice/isopentane bath, as we did in mouse orbital before^7,11,12^, consecutive 10 µm thick coronary sections were cut using a Leica microtome—CM1850 cryostat (temperature, − 25 °C, Leica Biosystems, Buffalo Grove, IL, USA) and mounted on adhesive slides (Poly lysine slides, cat#63700-w1, Roth, Germany). In each macro-section of three (e.g., a, b, or c in Fig. 1), the three serial histological sections to be for ABC immune staining, Histo-ELISA/HE, and Histo-ELISA without antibody control, as indicated in Fig. 1, were collected and kept at − 70 °C until processed.

Except for the occlusion of the left middle cerebral artery, the native control mice were subjected to the same processing as the infarct mice were treated.

### Immune staining

The sections were thawed to room temperature for 30 min. The right or left hemispheres of brains in the sections to be quantified were circled (diameter of 9 mm) with the hydrophobic barrier using PAP Pen (Cat No H-4000, Vector Laboratories, Inc. Burlingame, CA 94010, USA). In order to keep identical sizes and areas of the circle among the slides, a color standard circle with a diameter of 9 mm on the plastic plate was put under the slides to be stained, and used for a reference of circling. For IHC analysis, we used the well-established protocol^7,12^ in our laboratory.

The sections were immunostained using an avidin–biotin complex (ABC) method. After postfixation in 4% paraformaldehyde (pH 7.4), endogenous peroxidase activity potentially present in the brain was quenched by 10 min incubation with 0.3% hydrogen peroxide in PBS at room temperature. Slides were washed twice with PBS, and incubated for 30 min with a blocking medium of 10% goat serum (DAKO, Hamburg, Germany), 2% BSA (biotin free, Roth, Germany), and 1% swine serum (sc-2486, Santa Cruz Biotechnology) in PBS to block unspecific binding. Following 30 min incubation with biotinylated goat anti-mouse IgG antibody (Goat anti-mouse IgG-B, sc-2039, Human absorbed, Santa Cruz Biotechnology, USA) at dilutions of 1:300 with 2% BSA in TBS, 3 times' washing in PBS and incubation of streptavidin-conjugated HRP (1:100, DAKO, Hamburg, Germany) for 30 min were performed on the sections. Secondary to 4-time changes of washing in PBS, colorization was developed in peroxidase substrate solutions.

Antibody binding was visualized by adding DAB solution (Liquid DAB substrate, DAKO, Hamburg, Germany), and incubation for 5 min at room temperature for the slide of ABC immune staining (1st section in Fig. 1) or via 90 µl TMB solution (1-Step Ultra TMB-ELISA, Thermo science, USA) incubation for 4 min at room temperature for the slide of Histo-ELISA/HE (2nd section in Fig. 1).

As a blank control, on the other adjacent slide to the TMB applied one, the 3rd section in Fig. 1 was treated identically to that of the 2nd section, except that the biotinylated goat anti-mouse IgG antibody was replaced by the same volume of PBS.

### ELISA reader interpretation

After 4 min of coloration, 40 µl of the hydrolyzed substrate solution on the TMB applied slides were transferred onto microtiter plates in duplicate for each section. The Coloring reaction in each well was stopped with 120 µl of 1 M H_2_SO4, which had been filled beforehand. The plates were detected in a Tecan Sunrise ELISA reader for automated quantification of staining signal at 450 nm (reference wavelength 590 nm). An average of two well optical densities stands for the value of each section. The mean values of the three average optical densities from three sections spaced 2 mm apart were determined per animal. The slides were washed with water and prepared for the subsequent HE staining.

### HE staining and interest area determination

Following immune staining and washing, the sections (2nd section in Fig. 1) were stained with standard HE procedure, dehydrated, cleared, and coverslipped with Permount (Fisher Scientific, Schwerte, Germany). An × 2.5 objective lens (Axioscope; Carl Zeiss) was used to view the sections. Images were captured with an Axiovision digital camera system and recorded with a 2560 × 1920 pixel resolution. The left or right hemisphere areas in the sections of the brain were determined with Photoshop Software (Adobe Photoshop 5v) as we did before^7,11,13,14^.

### IHC score in immune staining slides

After coverslipping, the sections (first cryo-section, in Fig. 1) with positive staining (brown sedimentations) were photographed. The involved staining areas were determined with Photoshop Software, followed by IHC score evaluation, which is a measure to convert classic IHC into a more quantitative range. IHC score is based on staining intensity and percentages of the stained area in the hemisphere of the brain to be tested. Staining intensity is divided into four IHC categories reported: negative (0), weak (1 +), moderate (2 +), and strongly (3 +) stained. In each case, a histoscore was calculated as follows: IHC score = (1 × weakly stained relative area) + (2 × moderately stained relative area) + (3 × strongly stained relative area). The relative area is the proportion of the staining area to the whole-brain hemisphere area.

### Standard curve

A gradient serial of 3 times’ diluted mouse IgG solution (Chrom pure mouse IgG, whole molecule, Cat# 015-000-003, Jackson Immuno Research) in 4% BSA coating buffer (1.5% Na_2_CO_3_ and 0.293% NaHCO_3_) were prepared. Their concentrations are 840, 280, 94, 31, 10 and 0 ng/100 µl. 100 µl of the solution for each concentration was dropped into one of the 6 wells of 9 mm in diameter on the adhesive slide as mentioned before. The slide was allowed to dry at room temperature overnight.

To prepare the wells on the adhesive slides, the poly liner was peeled from the silicone gasket (Cat#S1810-25EA, Silicone isolators, Sigma) surface and put on a table with a stick-side up. The adhesive microscopic slide was placed atop and pressed onto the liner for a seal. The seal was inspected through the glass and the liner was pressed locally on the glass slide to remove any residual air bubbles.

**Figure 2:**
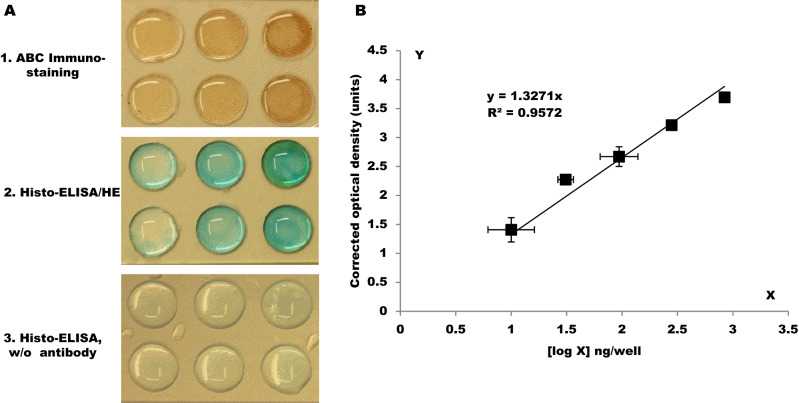
Coloration of IgG coated wells and standard curve. (**A**) Colorizations developed by DAB and TMB. Six wells in a slide, from top to bottom and right to left, gradient decreasing concentration solutions of mouse IgG were coated in sequence. The concentrations of 100 ml for the well coating are 840, 280, 94, 28, 10, and 0 ng/100 µl respectively. The first six-well slide (A1) was subjected to immunostaining and various intensity of brown colorization was achieved by DAB development. The second and third six-well slides (A2 and A3) underwent immunostaining and blue colorization was developed by TMB. The different intensities of blue color in each well were displayed just before transferring for ELISA interpretation. B. the standard curve was obtained from ELISA interpretation of A2. The linearity is shown in the standard curve (Pearson’s r = 0.9783, *P* < 0.000089). An EXCEL software was used to calculate the curve formula (y = 1.3271x). The y axis indicates the corrected optical density and the x axis the logarithm of coating weight—[log x] ng/well.

Parallel to the brain section staining, in the exact same way as we did in the brain sections above, we completed ABC immune staining, Histo-ELISA/HE staining and Histo-ELISA (w/o antibody) in three wells’ coated slides respectively (Fig. 2A). Cares were taken to ensure that the liquid on the slide surface was blotted before the application of TMB.

The plotted graph (Fig. 2B) was made, in which the logarithm of IgG coating weight—(log (x)) ng/well in x-axis was against the corrected optical density (y-axis), and a standard curve formula was made with aid of the software (Excel, Microsoft Office 2007).

We narrowed the range of concentration based on the trial test beforehand in which the gross maximum was determined from the sections of infarct brains and the minimum from those of the native intact brains.

### Parameters

In order to minimize the possible bias due to the difference between the IgG coating and brain tissue section background, the corrected values (corrected OD) were used to prepare the standard curve and for OD translation into IgG infiltration weight in the cerebral sections. The corrected values are the values measured subtracted the values from the corresponding adjacent slide of blank control (without antibody used) to balance the difference between the coating and brain sections.

IgG content (ng) per cubic millimeter (mm^3^) in brain tissue was determined by the ratio between weight translated from the optical density, by the reference of standard curve formula, and the corresponding section area multiplied by the section thickness (10 µm).

### Data analysis

Data are presented as mean ± SEM and analyzed using Student’s t-test (two-tailed) of Excel software (Office 2007 version) for comparison of means of two groups. The software SPSS (IBM Corp. IBM SPSS Statistics for Windows, Version 11.0., USA) was employed for analysis of correlation or multiple comparisons of means. A *P* < 0.05 was considered statistical significance.

## Results

### Sensitivity, limit, and standard curve

**Figure 3:**
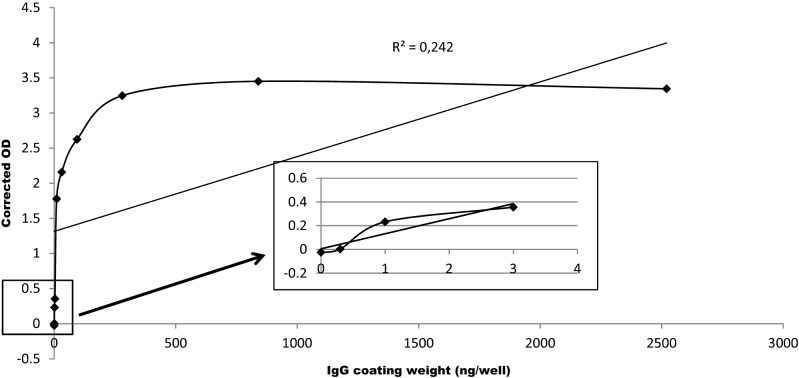
Analytical sensitivity tested using a coating of serial IgG dilution and corresponding corrected OD. Inset right is a high magnification of the box left.

It is not possible to measure the sensitivity in IHC assays because no reference analyte for tissue exists. Instead, the analytical sensitivity of IgG with Histo-ELISA was tested by diluting the IgG in the slides, in the fashion of standard curve preparation. In our trial test, a broader range of diluted mouse IgG (2520, 840, 280, 94, 31, 10, 3.0, 1.0, 0.3, and 0 ng/100 µl) was prepared and 100 µl of each concentration was coated in the wells of slides. Histo-ELISA staining in these diluted IgG coating slides yielded the results (Fig. 3). Sensitivity is the lowest amount of analyte in which the corresponding signals are distinguishable from the background or negative control, in other words, 0 in the corrected OD. In the present setup of the assay, the lowest amount of IgG in Fig. 3 is 0.3 ng/well (refer to the inset of Fig. 3), equivalent of 0.5 ng/mm^3^ in the section of tissue (9 mm diameter round area and 10 µm in thickness). The highest amount of analyte is a value in which the corresponding signal reaches saturated (Corrected OD can not increase anymore with an additional amount of analyte). It is ~ 1000 ng/well in the graph, equivalent of ~ 1600 ng/mm^3^ in the section of tissue. The limit of quantification is the range between the lowest and highest amount of analyte, which reflects 0.5–1600 ng/mm^3^ in the present setup. It is in the limit of quantification, that the analyte can be quantitatively determined with suitable precision and accuracy.

In addition, we found, in the trial test, the lowest amount of IgG in the section of saline-intact brains, which are equal to 10 ng/well of IgG coating in the density of colorized solution by native eyes, and the highest one, in the section of saline-infarct brains, to 840 ng/well. Therefore, we narrowed the standard curve mentioned, to an interval of 10–840 ng/well, and 0 ng/well serves as a control for the measurement (Fig. 2B). A higher linear correlation was displayed between the logarithm of IgG coating weight per well and corrected OD in the interval curve (Pearson’s r = 0.9783, *P* < 0.000089). The results are in line with those of ABC immune staining. The wells (Fig. 2A-2) in the slide of Histo-ELISA/HE with the TMB coloration demonstrate that from top to bottom and left to right, the blue color intensity decrease as IgG solution concentration is reduced (the last one is 0 ng/100 µl). In the contrast, in the slide of Histo-ELISA/HE, w/o antibody in Fig. 2A-3, the blue color intensity in the wells remains nearly unchanged – lower light blue color intensity. The brown color intensity developed by DAB, in the slide of ABC Immuno-staining in Fig. 2A-1, is comparable to that observed in the slide of Histo-ELISA/HE (Fig. 2A-1). As a negative control, the well (0 ng/100 µl) in absence of IgG displays very low color intensity. From the results, we can infer that the procedure and reagents used are highly specific and IgG coloration reveals a well dosage-dependent manner.

The optical density in these colorized TMB solutions in each well was measured in the ELISA reader and from these data, the standard curve was formed using software of Microsoft Office Excel 2007 (ref to Fig. 2B). The good linearity and dosage-dependent manner are reflected by the coefficient R^2^ = 0.9572. The formula in the standard curve was determined.

y = 1.3271(log(x)); where y = corrected optical density units and x = IgG coating weight (ng)/well. We used the formula y = 1.3271(log(x)) to translate the corrected OD detected in the cerebral sections into IgG content—ng per well cross the area. Thus, the amount of IgG infiltration present in sections of the left or right hemisphere in the brain was calculated out.

### Accuracy and comparison between Histo-ELISA and standard ABC staining

Accuracy is the closeness of agreement between the test result and an accepted reference value. Its assays can only be estimated and not fully determined since there are currently no IgG assays for direct IgG infiltration quantification widely accepted.

**Figure 4:**
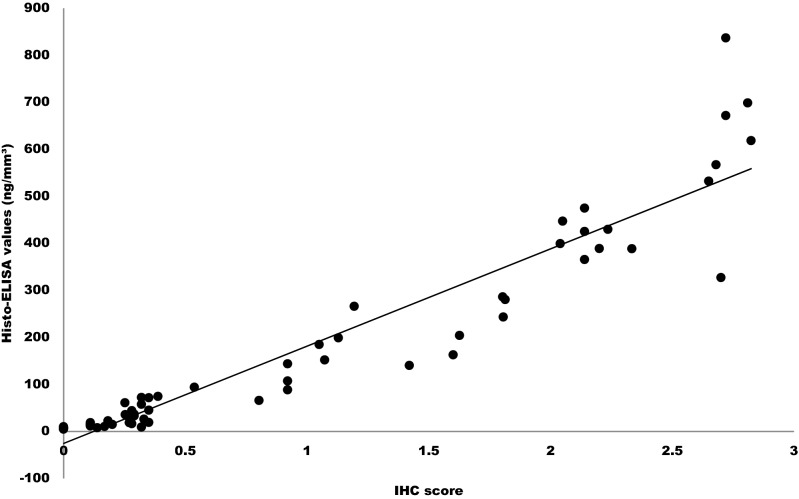
Accuracy tested on IgG measurements using Histo-ELISA and ABC immune staining. Statistic analysis resulted in statistical significant correlation. N = 66 pairs, Pearson’s r = 0.9436, *P* < 0.0001.

**Figure 5:**
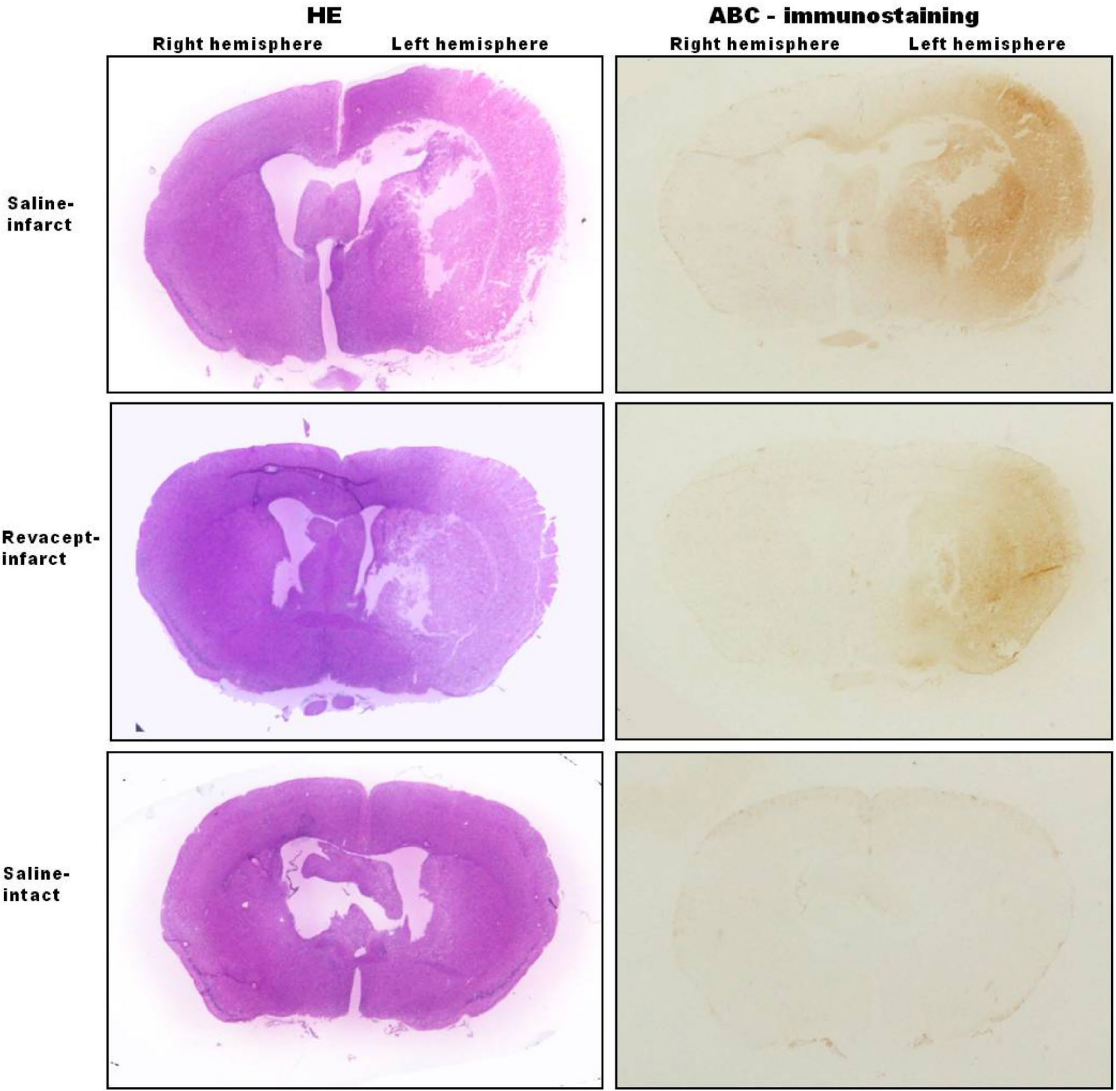
Brain morphological structures and IgG infiltration distribution. Brain structures were well shown after hematoxylin eosin staining (HE, left panels), and ABC immunostaining (ABC, right panels). Larger IgG infiltration area and higher intensity of brown color in brain sections in the stroke mice (saline-infarct) turned out smaller area and lower intensity of the color after treatment with revacept (revacept-infarct). In contrast, there were no obvious IgG extravasation distribution in the vehicle control mice (saline-intact).

The degree of closeness between the Histo-ELISA result and IHC score—an accepted reference value was tested by assessing the correlation between. In our study, we pooled the IHC scores of both left and right brain hemisphere in all the groups and compared to Histo-ELISA determined values of the corresponding adjacent sections. The resulting data, as indicated in Fig. 4, demonstrated a statistically significant correlation (Pearson’s r = 0.943635, *P* < 0.0001). Morphological structures stained with HE after Histo-ELISA evaluation or ABC immune staining in the adjacent brain sections were shown at the same time (refer to Fig. 5). The images show that there was a patch of necrosis, reflected by light staining areas of HE staining (see HE in the left panel of Fig. 5) of the left cerebral hemisphere from MCA occlusion mice. The high density of brown color covered the whole corresponding necrosis region of the left hemisphere in the saline-infarct group (larger IHC scores) and, to less extent (smaller IHC scores), in that of non-ischemic right hemisphere and saline-intact vehicle control mice in ABC immunostaining (right panel of Fig. 5). The high brown color intensity of IgG infiltration became lower and the distribution area smaller in brains after revacept treated in the stroke mice (revacept-infarct).

High brown color density in infarct regions or low one in the non-ischemic area of the section of ABC immunostaining was in agreement of respective larger and smaller values detected by Histo-ELISA. A strong correlation was evident between Histo-ELISA and standard ABC (Figs. 4, 5).

### Precision

To assess the variability of measurements’ precision, studies were based on the variation of Histo-ELISA data dots among the four groups. The four groups (0.00, 0–0.99, 1.00–1.99, and 2.00–2.99) were divided, based on the IHC score.

**Figure 6:**
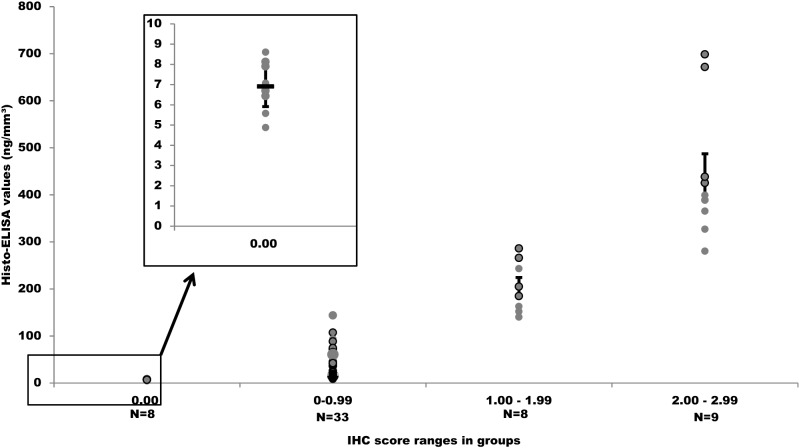
Precision analysis of Histo-ELISA value distribution in four groups. The four groups were divided according to the range of IHC scores. Inset up is high magnification of the box down. Cross bar and vertical line indicate means and SEMs respectively. Multiple comparisons with Dunn's resulting in statistical significantly difference between groups for all, *P* < 0.0001–0.0033.

The separation between the individual groups was tested by Dunn's multiple comparisons of SPSS. The results were shown that Histo-ELISA values of four groups were statistically significantly different between (for all, *P* < 0.0001–0.0033), and are well separated (Fig. 6). It must be stated, however, that the single dots in the intro-group of 0.00 were also distinguishable (inset in Fig. 6), and which indicates that a higher sensitivity of Histo-ELISA in comparison with that of IHC.

### Repetitive evaluation of IgG infiltration

To be able to trust a quantitative method, the method should be reproducible and not be affected by day-to-day variation. The adjacent sections in a serial or the sections derived from the same procedure treated animals should return the same results if reassessed at a later day. To determine the accuracy and repeatability of the procedure, we, in the third run assay, repeated the measurements with adjoining cerebral sections to the second one described above, and a new parallel set of the identical serial various concentration coating slides for the preparation of standard curve. The first run assay is a separate serial experiment, in which four groups of 8 animals served as saline-infarct, saline-intact, revacept-infarct, and revacept-intact group animals respectively. Two animals for each group were subjected to the same corresponding processing as described before and a parallel standard curve was completed with the same procedure.

The results, as shown in Tables 1 and 2, were in high similarity among three runs of measurements. The overall variation coefficient for three runs in different days was 0.045 in the values of corrected OD of the standard curve and 0.28 in the IgG infiltration in the brains, which reflects a rather high reproducible procedure of the technique over time. All three runs were compared using one-way ANOVA and Turkey post-test for multiple comparisons of SPSS, resulting in all *P* values of > 0.9668, and thus no significant difference between.

**Table 1:** Repeatablitiy testing on the IgG coating and the corresponding corrected OD measured with Histo-ELISA, tested on 3 different days.

Coating amount (ng/well)	First measument	Second measurment	Third measurment	C.V
	Corrected OD	Corrected OD	Corrected OD	
840	3.451451605	3.69975003	3.694842106	0.039
280	3.247368657	3.208879475	3.214252095	0.006
94	2.625345538	2.668849854	2.671199918	0.010
31	2.158066509	2.173491034	2.274800122	0.029
10	1.777813855	1.399525253	1.407700038	0.141
Av				0.045

The overall coefficient of variation for the 3 runs was 0.045, ranged from 0.006 to 0.14. Multiple comparison with Tukey HSD resulting in no statistic significance among the groups, for all, *P* > 0.9668.

**Table 2:** Mean values of IgG infiltration and coefficients of variation (CV) obtained from three runs of measurments in the adjacent cerebral sections of right or left hemispheres with Histo-ELISA on 3 different days.

Groups	First run			Second run				Third run		CV
	N	Mean (ng/mm^3^)	SEM	N	Mean (ng/mm^3^)	SEM	N	Mean (ng/mm^3^)	SEM	
Saline-intact right	2	28.799	59.911	3	38.824	8.545	3	34.537	12.457	0.148
Saline-intact left	2	58.214	9.817	3	38.896	10.820	3	27.499	9.894	0.374
Saline-infarct right	2	298.125	80.731	3	324.681	133.877	3	253.041	27.355	0.124
Saline-infarct left	2	506.843	20.481	3	507.371	67.589	3	441.491	33.888	0.078
Revacept-intact right	2	24.650	5.045	3	59.780	44.145	3	84.381	41.078	0.533
Revacept-intact left	2	15.700	0.652	3	25.349	14.045	3	39.172	12.811	0.441
Revacept-infarct right	2	193.935	48.308	3	178.749	42.561	3	85.583	40.606	0.384
Revacept-infarct left	2	240.918	46.732	3	193.048	40.390	3	179.863	66.034	0.157
Av										0.280

The overall coefficient of variation for the 3 runs was 0.28, ranged from 0.078 to 0.53. Multiple comparison with Tukey HSD resulting in no statistic significance among the groups, for all, *P* > 0.9678.

### Determination of IgG infiltration with Histo-ELISA and effectiveness of revacept

The results described above have shown Histo-ELISA is a rather high reliable quantitative assay. Such, we applied the method for the determination of IgG infiltration to investigate the effectiveness of revacept on stroke mice.

Twenty four hours after left MCA occlusion in the stroke mice (saline-infarct), the cerebral IgG increased 13 times as many as that of saline injected vehicle native control (saline-intact) in left hemisphere (507.4 ± 67.6 compared to 38.9 ± 10.8 ng/mm^3^, *P* < 0.01) and 8 times in right hemisphere (324.7 ± 133.9 compared to 38.8 ± 8.6 ng/mm^3^, *P* < 0.05). After treatment with 1 mg/kg revacept, the brain infiltrated IgG in revacept injected infarct mice (revacept-infarct) reduced twofold both in left hemisphere (507.4 ± 67.6 vs. 193.1 ± 44.4 ng/mm^3^, *P* < 0.01) and in right hemisphere (324.7 ± 133.9 vs. 178.8 ± 42.6 ng/mm^3^, *P* < 0.05), as compared to that of saline injected infarct mice (saline-infarct) (see Supplementary Fig. S1A). However, revacept did not exert the same impact on the vehicle native mice (revacept-intact) in the left hemisphere (saline-intact vs. revacept-intact; 38.9 ± 10.8 vs. 25.4 ± 14.1, *P* > 0.05) or in the right hemisphere (saline-intact vs. revacept-intact; 38.8 ± 8.6 vs. 59.8 ± 44.2, *P* > 0.05) (see Supplementary Fig. S1B).

## Discussion

Laboratory daily routine works on the path-histology resulted in the development of Histo-ELISA—a method for Quantitative Evaluation of Tissue Components in Locality. We have used a part of the method to quantify relative IgG evasculation in stroke and native mouse brain slices^7^. The parameter—optical density (O.D) per square millimeter (mm^2^) in the cerebral sections was used to compare IgG infiltrations from differently treated mice. The results show that the administration of Revacept (recombinant dimeric GPVI-Fc) results in a beneficial effect in the ischemic brain (reduction of IgG infiltration). But the index used in our previous experiments^7^ is only a relative value and not good enough to compare the profiles obtained from different laboratories or even in the same laboratory at different time periods, not to mention comparing the data from various detected methods—e.g. western blot or ELISA. Therefore, we improved and perfected the method we used before^7^ and developed a novel technique, Histo-ELISA, in expectation of an accurate, precise, and reproducible quantification method and universal parameters with which the data is intercomparable among different laboratories or different methods.

For the feasibility of Histo-ELISA, comparison with standard ICH—an accepted reference method, was performed. Reproducibility was assessed in a setup to resemble daily protocol. For all evaluated parameters, Histo-ELISA performance was either comparable to or better than the reference method. It was thereby demonstrated that Histo-ELISA is a quantitative, sensitive, accurate, and precise test with a rather high reproducibility.

In order to assess the validation parameters, determination of IgG in the cerebral sections from native and MCA occluded mice injected respectively with saline or revacept was performed. Parallel to the brain section evaluation, a serial gradient diluted IgG coated on the same type slides were detected in the exact same protocol for the standard curve. To narrow the range of the standard curve IgG concentrations, in our pre-experiments, the maximum concentration was determined with the ischemic sections from the saline-injected infarct mice and the minimum value with native intact sections from the saline-injected native mice (saline-intact) with aid of a wider range of IgG concentration coating slides (Fig. 3).

Optical density detected with Histo-ELISA in the cerebral sections was translated into the amount of IgG infiltration (ng/mm^3^), by the standard curve formula. The results were shown that treatment with revacept leads to a significant reduction of cerebral IgG infiltration in ischemic stroke (see Supplementary Fig. S1A). By using the technique, we demonstrated that revacept benefited local cerebral ischemia by reducing IgG extravasculation in the infarction zone. There were no affects on the non-ischemic brain of native mice (see Supplementary Fig. S1B). The results indicate that the data obtained from Histo-ELISA are in agreement with the data from the report^7^ before.

In order to compare between Histo-ELISA and the standard ABC immunostaining, the adjacent sections to the one of Histo-ELISA were objected to the ABC immunostaining, coupled with DAB coloration and IHC score evaluation. A close correlation was found in between (see Fig. 4). Morphological changes were reflected by the high intensity of brown color found in the left ischemia hemisphere of the brain and to less extent in the non-ischemic right hemisphere or intact brain from vehicle native mice (Fig. 5), which respectively correspond to the high or lower values obtained via Histo-ELISA with TMB coloration. Thereby, the results with Histo-ELISA or the standard ABC immunostaining are comparable between.

However, for assessment of the extent and intensity of the brown positive signal, which is used to quantify the standard ABC immunostaining, an arbitrary ‘weak, moderate and strong’, depending on how the assessor feels, is not appropriate^15^. Contrary to the well-documented subjectivity and intra- and inter-observer variability of manual, visual-based semiquantitative estimation of intensity or even of percent positivity^16,17^, the designed automated methods are immune to the consequences of fatigue and subjectivity faced with observers during signal intensity measurement. Target cell count or interest area image analysis can outperform human observers, certainly in terms of precision and quantitative reproducibility^18^. Coloration with TMB instead of DMB facilitates automatic evaluation with ELISA reader as in the technique of Histo-ELISA. The optical densities were then translated to the target protein contents in the tissue via a standard curve obtained with a parallel slide coated with various quantity. The more precise values were given out (Fig. 6), which allows comparisons with other various laboratories or different methods such as ELISA or western blot. Concerning the methods—ELISA or western blot, Histo-ELISA makes up for the shortcomings of ELISA in which the morphological data related to the protein distribution or accompanied structure changes is lacking. In addition, rather high reproducibility of the technique is also inferred (refer to Tables 1 and 2).

It must be stated, however, that like immunohistochemistry and conventional ELISA in principle, accurate results of Histo-ELISA are dependent on the specificity of antibodies and standardization of pre-analytical factors. Application range (analyte content in tissue) is limited by the highest and lowest dosage of the standard curve. In our setup experiment, the ELISA reader could read the IgG of 0.5–1600 ng/mm^3^ contained in the tissue and accurate results would precisely give out within this rang. If broadening the application rang, practical features of Histo-ELISA can be used to tune the assay for the target proteins, e. g., adjust section thickness or proportion of coloring solution to the stop solution of H_2_SO_4_. If the protein of interest is of low contents, it is possible to increase the section thickness or a higher proportion of coloring solution before interpretation by ELISA reader to magnify the signal and vice versa.

Although the main limitation to morphologic diagnosis is variability between pathologists, microscopic morphology remains the gold standard in current diagnostic pathology^19^. It is encouraging that we are witnessing a transformation in pathology as a result of the widespread adoption of whole slide imaging in lieu of traditional light microscopes. Depicting microscopic pathology characteristics digitally presents new horizons in pathology^20^. The introduction of artificial intelligence (AI) in the pathology domain, at least in the morphological analysis of tissues and cells, with the help of digital pathology equipment varying from microscopic cameras to whole slide imaging scanners, morphology-based automated pathologic diagnosis has become a reality^19^. Our method mentioned conforms to the trend of digital pathology, which supports the AI by transforming the signal of biomarkers into a digit.

This method, therefore, is reliable with rather high reproducibility. Its application includes but not limited to, protein detection. Quantification of interest cells can also be performed by the use of this method if the appropriate standard curve is used. Its application will facilitate the development of AI pathology.

## Supplementary information

Supplementary Figure.
