# Supplementary material for: Histo-ELISA technique for quantification and localization of tissue components
Source: Sci Rep. 2020 Nov 16;10:19849. doi: 10.1038/s41598-020-76950-1 (PMC7669848; doi:10.1038/s41598-020-76950-1)
Supplement: Supplementary file 1 — Supplementary Figure. [file 41598_2020_76950_MOESM1_ESM.docx]

Supplementary information

A Histo-ELISA technique for quantification and localization of tissue components

Zhongmin Li^1*^, Silvia Goebel^1^, Andreas Reimann^1^ & Martin Ungerer^1^

^1^Advancecor GmbH, 82152 Martinsried, Germany.

*, Correspondence and requests for materials should be addressed to Z. L. (email: [li@advancecor.com](mailto:li@advancecor.com))

Figure S1

**Figure S1. Mouse IgG extravasation quantification in the brains. (A) IgG infiltration in brains of the stroke mice. IgG infiltration contents were multipled eightfold in right brain hemisphere and thirteen folds in left brain hemisphere after 24 hrs of occlusion in left middle cerebral artery, with contrast to those of vehicle saline-intact control. Treatment with revacept (1 mg/kg) cut down IgG extravasation by nearly 50% in right hemisphere and 60% in left hemisphere of stroke mouse. (B) IgG infiltration in brains of the native vehicle mice. On the contrary to observation of revacept treatment in the stroke mice, revacept did not affect the IgG infiltration in the vehicle intact mice. There was no significant difference between saline-intact and revacept-intact groups both in right and left brain hemispheres. The means of IgG infiltration contents in the brain are expressed as ng per cubic millimetre ± SEM, n = 3 per group. #, indicates P<0.05 and ##, P<0.01, vs. saline-intact; *, p<0.05 and **, p<0.01 vs. saline-infarct.**
